# Identification of the occurrence and pattern of masseter muscle activities during sleep using EMG and accelerometer systems

**DOI:** 10.1186/1746-160X-5-7

**Published:** 2009-02-11

**Authors:** Hidehiro Yoshimi, Kenichi Sasaguri, Katsushi Tamaki, Sadao Sato

**Affiliations:** 1 Department of Craniofacial Growth and Development Dentistry, Research Institute of Occlusion Medicine, Research Center of Brain and Oral Science, Kanagawa, Japan; 2 Oral and Maxillofacial Rehabilitation, Kanagawa Dental College, 82 Inaoka-Cho, Yokosuka, Kanagawa, Japan

## Abstract

**Background:**

Sleep bruxism has been described as a combination of different orofacial motor activities that include grinding, clenching and tapping, although accurate distribution of the activities still remains to be clarified.

**Methods:**

We developed a new system for analyzing sleep bruxism to examine the muscle activities and mandibular movement patterns during sleep bruxism. The system consisted of a 2-axis accelerometer, electroencephalography and electromyography. Nineteen healthy volunteers were recruited and screened to evaluate sleep bruxism in the sleep laboratory.

**Results:**

The new system could easily distinguish the different patterns of bruxism movement of the mandible and the body movement. Results showed that grinding (59.5%) was most common, followed by clenching (35.6%) based on relative activity to maximum voluntary contraction (%MVC), whereas tapping was only (4.9%).

**Conclusion:**

It was concluded that the tapping, clenching, and grinding movement of the mandible could be effectively differentiated by the new system and sleep bruxism was predominantly perceived as clenching and grinding, which varied between individuals.

## Background

Quality of sleep is strongly associated with somatic health and activity of the body. During sleep, many physiological events occur, such as sleep talking, sighing, swallowing, and bruxing along with decreased skeletal muscle activity, heart rate, body temperature and blood pressure [1]. Bruxism sometimes interferes with sleep quality. Sleep bruxism is reported to be a common phenomenon in humans and many studies have shown that bruxism can harm the dentition, its supporting structures and the temporomandibular joint (TMJ) [2-8]. Many bruxers are not aware of their behavior, and not all bruxers make noise that bed partners might notice. The definition of "bruxer" is based upon patient reports of a history of tooth-grinding occurring more than three times a week for at least six months, as attested by their sleep partners [6,7]. In addition, bruxers exhibited tooth wear, with orofacial jaw muscle fatigue, tenderness or pain or masseter muscle hypertrophy. Recently, we studied the prevalence of bruxism in the general adult population using a custom-made color-stained plastic sheet, the BruxChecker, on the maxillary dentition overnight and found that occlusal contacts where the color was ground off were seen in the majority of subjects, indicating sleep bruxism [9].

There is no scientific evidence that bruxism is a type of disease or abnormal function, however certain conditions which are caused by bruxism seem to be non-physiological phenomena. Rhythmic masticatory muscle activity does not disrupt nocturnal sleep, further suggesting that this motor activity is a natural activity occurring during sleep [8]. Lavigne et al. reported that the patients who have temporomandibular disorder (TMD) are sometimes conscious of the existence of sleep bruxism and they presented evidence to support the positive correlation coefficient between clinical symptoms of TMD and sleep bruxism. The influences of bruxism activity on TMD are not fully established [1].

The diagnosis and treatment planning of bruxism is becoming more relevant in dentistry, due to many degenerative oral diseases that seem to be related to excessive biomechanical load exerted by the strong masticatory muscle activities during bruxism. In clinical dentistry, practitioners must be aware of the criteria by which to distinguish patients who brux from those who do not. In this context, it is necessary to define the bruxism described as the physiological limit of muscle activity during sleep, in order to distinguish it from the non-physiological range of bruxism activity.

Previous sleep researches have shown the presence of various types of sleep bruxism. Phasic/rhythmic (more than 3 bursts), tonic (more than 2 seconds over burst), mixed (rhythmic+tonic) types [1,10], or steady-state and rhythmic clenching, grinding, and tapping [11]. Various bruxism detecting methods have been proposed. Polysomnography [12-17] and portable EMG [18-21] were used for measuring sleep bruxism. In addition to these, stent [22], splint [23], and splints that involves a piezo-electric element [11,24], were introduced as a bruxism-observing technique. Stent or splint techniques may increase or decrease activity. In these methods, the devices may influence bruxism activity due to alteration of vertical dimension, therefore it is not clear whether the data from these systems is specific or not. The ambulatory EMG (portable EMG) is adaptable to daily life, but the system is still not satisfactory due to the presence of considerable noise from the environment. Sleep laboratory systems, which include electromyography (EMG), electrokinesiography (EKG), electroencephalograpy (EEG), and audio system are precise, but the mental and physical stress from the laboratory environment should not be neglected. In this context, the actual status of bruxism activity during sleep is not exactly known, and there is no consensus concerning the amount and type of bruxism activity needed to define a certain type of event.

It is difficult to distinguish between the different activities by the electromyography (EMG) system alone. In this study, a newly developed method that may be useful to assess bruxism, which involves measuring mandibular movement during sleep, was applied to define different types of bruxism activities.

The purposes of this study were to investigate whether it is possible to differentiate the pattern of sleep bruxism using a newly developed simple device and to determine the distribution of the different types of bruxism activity. Attempt to establish the physiological range of bruxism activity was also considered.

## Materials and methods

In this study, 19 volunteers (healthy and young post graduated student and dental college students. 16 males and 3 females, aged 28.5 ± 5.8 years) consented to have their sleep bruxism activity analyzed. We recruited them unintentionally and they were not bruxer. The experimental design, procedures and tasks were carefully explained to the volunteers prior to starting the experiment. Each volunteer slept for the entire night with a bruxism-monitoring system in the sleep laboratory of Kanagawa Dental College. Experimental procedures were approved by the Human Ethics Committee of Kanagawa Dental College. We obtained informed written consent from all subjects, and we advised them of their right to discontinue the experiment at any time.

**Figure 1:**
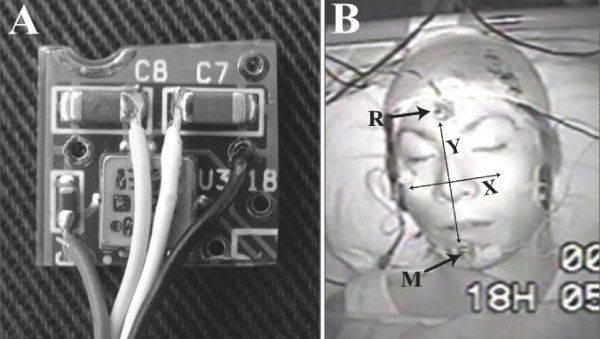
**Panel A shows the ACC used in this study**. Panel B shows the attachment sites of the reference ACC (R) and measurement ACC (M). Surface electrodes were located in areas of right and left masseters.

Self-adhesive surface electrodes were placed over the masseter-muscle on a vertical line between the zygomatic arch and the inferior border of the mandible (Fig. 1). Accelerometers were fastened on the forehead as a reference and on the middle point of the chin concavity of the mandible with vinyl polysiloxane and adhesive material. The muscle activity of maximum voluntary contraction in the volunteers was measured 30 minutes before they went to sleep in order to compare it with actual bruxism activity. To establish a relative level of contraction before the sleep bruxism recording, each subject performed at least 3 times intercuspal-position clenches that were less than 5 sec in duration at a 100% maximum voluntary contraction (MVC) effort. The initial MVC data for each subject were used to normalize all subsequent data so that all EMG signal could be reported as a percentage of the maximum (100%) signal.

The new monitoring system of sleep bruxism consisted of a 2-axis accelerometer (ACC, ADXL202E, Analog Devices Co. Ltd, Norwood, MA, USA), an electroencephalogram to measure sleep stage (EEG, Poly Mate AP1124, TEAC Co. Ltd., Tokyo, Japan) and EMG (EMG, SN 700, Techno Science Co. Ltd., Tokyo, Japan). An infrared video camera (Infrared LED CCD camera, KM-033, Koike Musen Denki Co. Ltd., Tokyo, Japan) recording system which had a time-lapse video cassette recorder (TLV-3060, Daiwa Co. Ltd., Tokyo, Japan) was used for monitoring sleep condition. Laser Doppler flowmetry (CDF-2000, Cyber Med, OAS Co. Ltd., Tokyo, Japan) was used to monitor blood-flow changes. We checked the reactive validation of bruxism-analyzing software (G1 System Co. Ltd., Tokyo, Japan) for body movement through infrared video camera and EMG data. Various kinds of noises were eliminated from raw data and identified the existence of mandibular reaction in the low muscle activity layer.

In this study, the criteria for bruxism activity were as follows: EMG threshold level was over 5% of activity, minimum time length of bruxism episode was 250 msec of muscle burst in the case of tapping and over 500 msec of burst in the cases of clenching and grinding, and minimum inter-episode time was more than 3 sec. Before measuring jaw movements, coefficient calibration through calibration voltage and scale value (physical set value) was calculated. Both calibration voltage and scale value to terminus point 2 and origin point 1 were established. We formulate first degree equations; procure inclinations and equations with canceling offset voltage (DC component). We obtained coefficient calibration data in this way.

**Figure 2:**
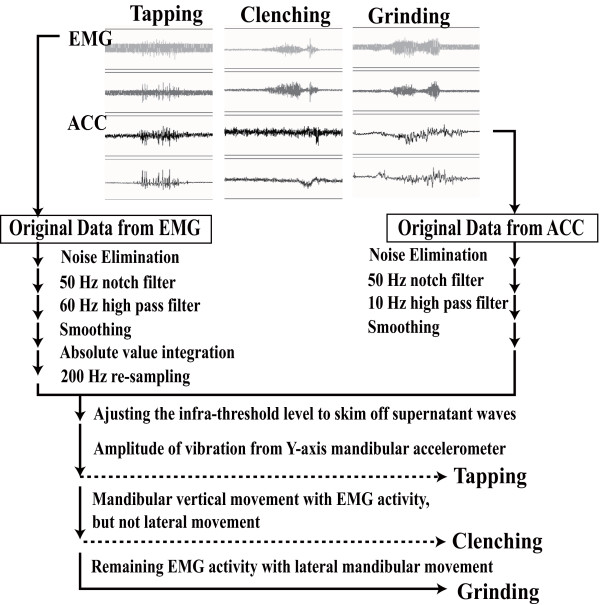
**Block diagram of data recording and analyzing system**. Tapping activity could be separated from raw data based on rhythmic, sharp and short integral EMG activity and Y axis movements. The clenching activity was separated from grinding activity based on the long continuous muscle bursts with no or small deviation of the Y axis, and residual grinding activity showed long continuous muscle bursts with mandibular movement in the Y axis.

Figure 2 shows a block diagram of the data recording and analyzing sequence is presented. Briefly, the original raw data from EMG and ACC had noise elimination using a 50-Hz notch filter and a 60-Hz high-pass filter, followed by smoothing and absolute-value integration. Step-by-step categorization of the assembled data provided different bruxism patterns. First, tapping activity was categorized in order to eliminate it from the raw data since tapping was most clearly recognizable and distinctive from other activities. Tapping movement was characterized by rhythmic, sharp and short integral EMG activity as well as Y-axis movements. The correlation coefficient of standard tapping wave shape was used to eliminate data that did not coincide with numerical values. In addition to these processes, the amplitude of vibration was calculated according to the following equation to exclude huge data.

J = Y × amplitude magnification

Y = (Hm + s.d.) × 2

Where J is the amplitude of vibration, Hm is the average of amplitude of vibration and s.d. is the standard deviation. Clenching activity was characterized by long continuous muscle bursts in EMG data with little or no deviation in XY-axis. The remaining EMG activity with long continuous muscle bursts and mandibular movement in the XY-axis was considered as a grinding pattern.

**Figure 3:**
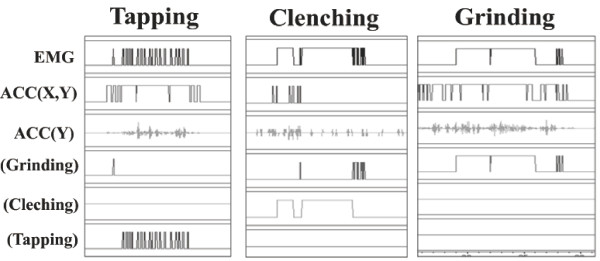
**Characterization of different patterns of bruxism activities**. Combined analysis of EMG and ACC showed that tapping was a rhythmic muscle activity with Y-axis movement, clenching was strong muscle activity with no Y-axis movement, and grinding was muscle activity with X and Y movement.

After setting up analyzing software, we checked the reactions through awakening voluntary basic movement and video recorder data of all volunteers. Basic test movements were carried out for tapping, small range right and left side grindings, wide range right and left side grindings, maximum muscle contraction (MVC) clenching with and without slight lateral movement, protrusion-retrusion. Figure 3 indicates the coincidences of analyzing software reactions and voluntary awaking jaw movements. It was realized that small muscle activity (under 5 %MVC) were easily smeared with noises and the number of events went to exceptional numbers.

### Statistical Analysis

One-way ANOVA and Tukey HSD test were used to establish significance for variables on each of the three types of bruxism activity, grinding, clenching, and tapping. Statistical significance was evaluated at P < 0.05. The statistical analyses were carried out using the Statistical Package for SPSS (version 13.0).

## Results

Using the newly developed system, Bruxism was assigned to three types; grinding, clenching and tapping. The distribution of different patterns of bruxism activity showed that clenching and grinding activities were more predominant, whereas tapping activity was not highly prevalent during sleep (Table 1, 2). Muscle activities (%MVC) were greater in grinding (59.6%) than in clenching (35.6%), while tapping activity was very low (4.9%). Calculation of occurrence of events and length of event also indicated that clenching and grinding were the predominant bruxism activities (Table 2). Sleep bruxism was constituted by 32.3% of grinding, 43.3% of clenching, and 24.4% of tapping activities based on the count of events; whereas 56.8% of grinding, 37.4% of clenching, and 5.8% of tapping were registered based on the length of events per hour.

**Table 1:** Distribution of muscle activity (%MVC) in different types of sleep bruxism

	Muscle activity (%MVC)			
	
	Mean	s.d.	Min-Max	(%)
Grinding	32.8	37.7	3.3 – 115	(59.5)
Clenching	19.7	23.4	0.3 – 106	(35.6)
Tapping	2.6	3.4	0.3 – 15.5	(4.9)
Total	55.1	58.4		(100)

**Table 2:** Distribution of event number, event length in different types of sleep bruxism

	Event number (/hour)				Event length (sec/hour)			
		
	Mean	s.d.	Min-Max	(%)	Mean	s.d.	Min-Max	(%)
Grinding	6.5	3.4	1.8–15.5	(32.3)	61.3	45.3	17.0–160.0	(56.8)
Clenching	8.7	4.7	1.5–18.9	(43.3)	40.4	51.7	1.60–211.5	(37.4)
Tapping	4.9	3.6	0–10.4	(24.4)	6.3	3.5	1.2–12.7	(5.8)
Total	20.1			(100)	108.0	90.4		(100)

**Figure 4:**
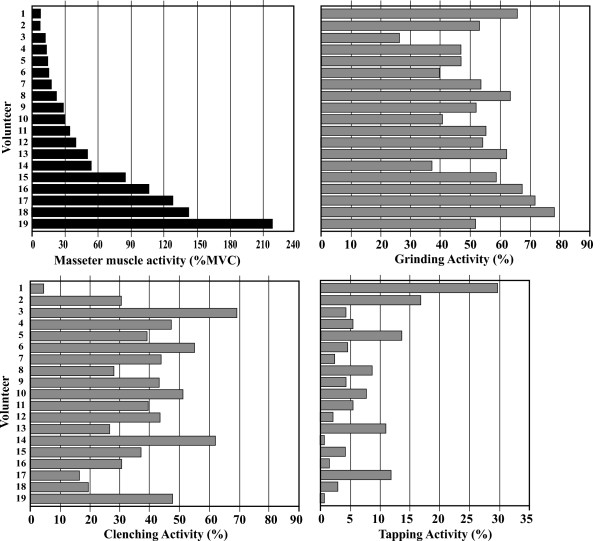
**Distribution of muscle activity (%MVC) into the different patterns of bruxism**. Variation of muscle activity (%MVC) in volunteers was observed. There was a tendency that subjects who had higher muscle activity showed relatively high grinding activity and lower muscle activity (%MVC) subjects showed relatively high clenching or tapping activities.

Fig. 4 shows the distribution of masseter-muscle activity (%MVC) and percent activity of grinding, clenching and tapping in each volunteer. A wide variation in masseter-muscle activity (%MVC) was observed. Subjects with higher muscle activity, such as volunteers 17 and 18, tended to show a relatively high grinding activity, while clenching and tapping activities were relatively low. In contrast, subjects with lower muscle activity (%MVC), such as volunteers 1 and 2, showed relatively high tapping activity.

**Figure 5:**
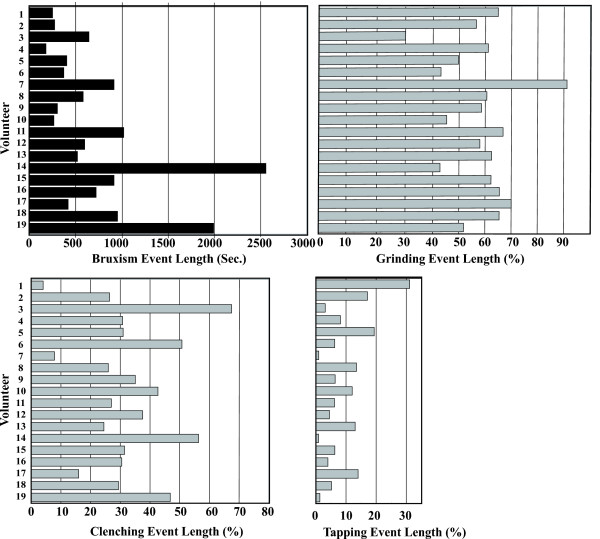
**Distribution of bruxism event length into the different patterns of bruxism**. There was a tendency for subjects who had long bruxism event duration to show increasing grinding event duration and decreasing clenching and tapping event durations.

Comparisons of the duration of bruxism-events demonstrated that individuals who had high muscle activity (%MVC) also tended to show long event duration similar to volunteers 18 and 19, whereas individuals with moderate muscle activity (%MVC) showed relatively long event duration such as volunteers 14 (Fig. 5).

**Figure 6:**
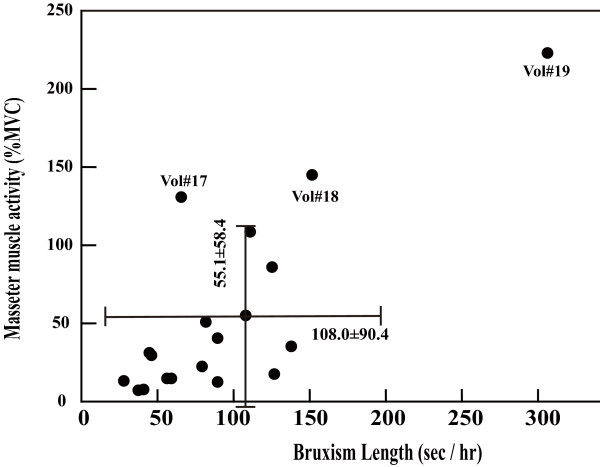
**Relationship between the muscle activity (%MVC) and the bruxism length (sec/hour) duration**. Majority of the volunteers were displayed in the lower left quadrant which means that muscle activity (%MVC) and bruxism event duration were not as high as in the volunteers.

Fig. 6 shows the relationship between the masseter-muscle activity (%MVC) and bruxism-event duration. The majority of volunteers are plotted in the lower left quadrant, indicating that the muscle activity (%MVC) and bruxism-event duration were not as high as the average values, 55.1 ± 58.4 (%MVC) and 108.0 ± 90.4(sec/hour), respectively. Seventy-nine percent of volunteers were within one standard deviation, while the values of volunteers 14, 17, 18 and 19 were out of the average range.

## Discussion

The definition of bruxism has evolved to include different behavioral mandibular movements such as grinding, clenching, and tapping. In this study, we developed a new analyzing system of bruxism and analyzed the behavior of sleep bruxism in 19 volunteers. The new analyzing system of bruxism has two major advantages. First, the combined system of EMG and Acc provides clear and easy distinction between real bruxism activity and other activities, such as the noise from body movements. Second, ACC analysis offers an effective and reliable way to differentiate the grinding, clenching and tapping activities. ACC packaging itself is very small and light (5 mm long, 5 mm wide, 2 mm thickness, under 1 g weight). Precise data can be gathered naturally.

The combined analysis of EMG and ACC provided distinctive patterns: rhythmic muscle activity with Y-axis movement as tapping type, strong muscle activity with no Y-axis movement as clenching type, and muscle activity with XY movement as grinding-type bruxism. The bruxism pattern in individuals during sleep varied widely with a combination of different mandibular movements. We still do not know how and when the different types of bruxism occur. Some individuals showed higher EMG activity than maximum voluntary clenching. This was also unexpected and it is not clear why such strong activity occurs.

Our study indicates that two types of bruxism were dominant, grinding and clenching. There was tendency that higher muscle activity was in grinding than that in clenching, especially in volunteers who brux strongly, although the length and events of clenching and grinding were not significantly different.

The results show that individual muscle activity (%MVC) had a wide distribution from 223.0 %MVC to 7.20 %MVC (Fig. 4). It was also demonstrated that muscle activity predominantly consisted of grinding and clenching activities. Tapping activity in bruxism was low relative to the grinding and clenching activities. Although we were still unable to fully define which level of bruxism activity can be considered as a diagnostic parameter to distinguish between the normal range of bruxism activity and bruxer or non-physiological activity, a normal range of bruxism activity can be proposed in which the average masseter-muscle activity (%MVC) and bruxism-event duration are 55.1 ± 58.5 (%MCV) and 108.0 ± 90.4 (sec/hr), respectively. Seventy-nine percent of the volunteers were included within these ranges.

Whereas the duration of tooth contact during parafunctional activity is fleeting in nature, an average episode of sleep bruxism may last as long as 4–5 seconds with the average rate of both grinding and clenching activities about 40 seconds per hour (Table 2). The more severe the sleep bruxism, the longer the teeth stay in contact with relatively high muscle activity (Fig. 6), resulting in larger sustained forceful muscle contraction.

## Conclusion

The innovative bruxism-analyzing system developed using EMC and ACC easily differentiates the three different bruxism patterns: grinding, clenching, and tapping. Sleep bruxism activity predominantly consisted of clenching and grinding, which varied between individuals. Seventy-nine percent of the volunteers were included within average ranges of 55.1 ± 58.4 (% MCV) and 108.0 ± 90.4 (sec/hr).

## Competing interests

The authors declare that they have no competing interests.

## Authors' contributions

HY collected the data from volunteers at the sleep laboratory and participated in the analysis of raw data of EMG, EEG, and ACC. KS participated in the development of new analyzing system of sleep bruxism using EMG and ACC. KT participated in collecting the data from the sleep laboratory together with HY and helped to construct research design. SS participated in the design of the study and coordinated the drafting of the manuscript. All authors have read and approved the final manuscript.

## Consent

Written informed consent was obtained from our volunteers for publication of this clinical report and the accompanying images. A copy of the written consent is available for review by the Editor-in-Chief of this journal.
